# Clinico–Pathological Features of Diffuse Midline Glioma, H3 K27-Altered in Adults: A Comprehensive Review of the Literature with an Additional Single-Institution Case Series

**DOI:** 10.3390/diagnostics14232617

**Published:** 2024-11-21

**Authors:** Giuseppe Broggi, Serena Salzano, Maria Failla, Giuseppe Maria Vincenzo Barbagallo, Francesco Certo, Magda Zanelli, Andrea Palicelli, Maurizio Zizzo, Nektarios Koufopoulos, Gaetano Magro, Rosario Caltabiano

**Affiliations:** 1 Department of Medical and Surgical Sciences and Advanced Technologies “G.F. Ingrassia”, Anatomic Pathology, University of Catania, 95123 Catania, Italy; giuseppe.broggi@phd.unict.it (G.B.); sere.salzano@gmail.com (S.S.); mariafailla97@gmail.com (M.F.); g.magro@unict.it (G.M.); rosario.caltabiano@unict.it (R.C.); 2 Department of Neurological Surgery, Policlinico “G. Rodolico-S. Marco” University Hospital, 95121 Catania, Italy; gbarbagallo@unict.it (G.M.V.B.); francesco.certo@unict.it (F.C.); 3 Pathology Unit, Azienda USL-IRCCS di Reggio Emilia, 42123 Reggio Emilia, Italy; andrea.palicelli@ausl.re.it; 4 Surgical Oncology Unit, Azienda USL-IRCCS di Reggio Emilia, 42123 Reggio Emilia, Italy; maurizio.zizzo@ausl.re.it; 5 Second Department of Pathology, Medical School, National and Kapodistrian University of Athens, Attikon University Hospital, 15772 Athens, Greece; nkoufo@med.uoa.gr

**Keywords:** diffuse midline glioma, diffuse intrinsic pontine glioma, adult, H3 p.K28M, H3 K27-altered

## Abstract

**Background:** Diffuse midline glioma (DMG), H3 K27-altered, is a WHO grade 4 malignant glioma located at midline structures, including the thalamus, brainstem and spinal cord. While H3 K27-altered DMG is more common in pediatric age in which it shows a uniformly aggressive clinical behavior, its occurrence is relatively unusual among adults, and its clinico–pathological and prognostic features are not fully characterized in this age group. **Methods:** In this present paper, a review of the literature, including all cases of adult H3 K27-altered DMG published from January 2010 to December 2023 was performed, and the following clinical parameters were evaluated: sex, age (median and range), anatomic site, median follow-up, leptomeningeal dissemination, local recurrence and treatment. In addition, the clinico–pathological features of three additional adult cases from our retrospective series were also reported and discussed. **Results:** All tumors from our series exhibited a high-grade morphology with brisk mitotic activity; microvascular proliferation and necrosis were seen only in one case. The immunohistochemical loss of H3 K27me3 along with diffuse and strong immunoreactivity for H3 K27M was found in all cases, leading to the diagnosis of H3 K27-altered DMG. **Conclusions:** The literature review showed that adult H3 K27-altered DMG more frequently occurred in males aged between 18 and 40 years. The thalamus was the most affected site, followed by the brainstem and spinal cord, in both sex groups. Adult tumors exhibited less aggressive clinical behavior, with leptomeningeal dissemination and local recurrence reported in only 23.78% and 37.75% of cases, respectively.

## 1. Introduction

K27 and K28 refer to specific lysine residues in the histone H3 protein, which plays a critical role in the regulation of chromatin structure and gene expression. In the context of histone H3-altered glioma, these residues are involved in mutations/molecular alterations that contribute to tumor development and growth. According to the 5th edition (2021) of the WHO Classification of Central Nervous System (CNS) Tumors, diffuse midline glioma (DMG), H3 K27-altered, is a pediatric-type, infiltrative, high-grade glioma arising from midline structures that exhibits H3 p.K28M mutation and usually either an H3 c.83A>T p.K28M substitution in one of the histone H3 isoforms, aberrant overexpression of EZHIP or an EGFR mutation (CNS WHO grade 4) [1,2,3,4,5]. DMG is a typical tumor of the pediatric age, but it may less frequently occur in adults [6]. The most common locations are the brain stem, the thalamus and the spinal cord [6]. Although the presence of brisk mitotic activity, microvascular proliferation and necrosis are frequent histologic features of DMG, they are not necessary for diagnosis, and the WHO assigns a grade 4 to this entity regardless of its morphology if one of the abovementioned molecular criteria is found [1,5].

As H3 p.K28M mutations have rarely been reported in other tumors exhibiting different clinico–pathological features and prognoses than DMG, including ependymomas, Pilocytic astrocytomas, pediatric-type diffuse gliomas and gangliogliomas, both the C-IMPACT-NOW Working Committee and WHO have emphasized the fact that the designation of “DMG, H3 K27-altered” should be applied only to those gliomas that are diffuse, midline, and astrocytic in morphology and that meet the essential molecular criteria for diagnosis and not to all H3 p.K28M mutant CNS tumors. Immunohistochemical analyses for H3 K27M, H3 K27me3 and EZHIP status may be used to confirm this diagnosis [1,5,7,8,9]. H3 K27-altered DMGs variably exhibit aberrant nuclear expression of p53, suggesting a TP53 mutation and loss of α-thalassemia/mental retardation syndrome x-linked (ATRX) expression in approximately 50% and 15% of cases [1,5,8,10]. On magnetic resonance imaging (MRI), DMGs in the conventional presentation have their epicenter in the pons, often asymmetrically, with frequent encasement of the basilar artery [11]. The prognosis is poor, and the 2-year survival rate is less than 10% [1,10].

As DMGs are relatively unusual in adults, limited epidemiological data are currently available in the literature; the incidence is estimated to be 2.32 cases per 1 million persons/year in people older than 20 years, with no sex predilection [6,7,12]. Most patients show the classic triad: cranial nerve palsy, long tract signs such as pyramidal tract impairment and ataxia. Upon MRI, DMGs classically have their epicenter in the pons, often asymmetrically, with frequent encasement of the basilar artery [6,7,12]. In this present study, we systematically reviewed all cases of DMG diagnosed in adult patients, and we additionally report three further adult cases from our institution, whose clinico–pathological and immunohistochemical features are emphasized.

## 2. Materials and Methods

The studies involving human participants were reviewed and approved by the local ethics committee, Catania 1 (CE 165/2015/PO). The patients provided written informed consent to participate in this study.

All cases with a pathologic diagnosis of H3 K27-altered DMG were retrospectively retrieved from the Pathology Archive of the Department of Medical, Surgical Sciences and Advanced Technologies “G.F. Ingrassia” of the University of Catania. Age > 18 years was the only inclusion criterion adopted. Three cases of adult patients with H3 K27-altered DMG were found and included in the study. Clinical data were retrieved from the original pathology reports. Hematoxylin and eosin (H&E)-stained sections were reviewed by two pathologists for diagnostic confirmation. The following immunohistochemical (IHC) slides, with appropriate positive control, were evaluated for all cases: glial fibrillary acidic protein (GFAP), isocitrate dehydrogenase-1 (IDH1) (p.R132H), ATRX, p53, H3 K27M, H3 K27me3 and Ki67.

A literature review was performed using PubMed/MEDLINE, searching for all published English language cases of adult (beyond the age of 18 years) H3 K27-altered DMG from January 2010 to December 2023. The following Medical Subject Headings (MESHs) were used: diffuse midline glioma, diffuse intrinsic pontine glioma, adult, pons, thalamus, medulla oblongata, midbrain, spinal cord, brainstem, H3 p.K28M mutation and H3 K27-altered. Both case series and single case reports on this topic were included in our review. The following clinical parameters were evaluated: sex, age (median and range), anatomic site, median follow-up, leptomeningeal dissemination, local recurrence and treatment.

## 3. Results

### 3.1. Case Series

Table 1 summarizes the clinico–pathological and immunohistochemical features of our cases.

**Figure 1:**
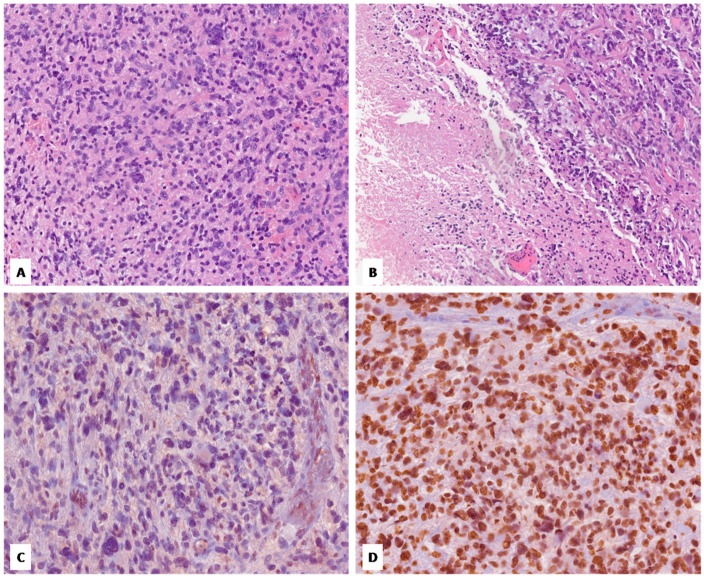
Case no. 1. (**A**) Histological examination shows a hypercellular diffuse glioma with astrocytic morphology, exhibiting severe nuclear pleomorphism with a multinucleated giant cell component. (**B**) Foci of tumor necrosis are seen. (**C**,**D**) Neoplastic cells show loss of nuclear staining for H3 K27me3 (**C**) and are strongly and diffusely positive for H3 K27M (**D**) (**A**–**D**, original magnifications 300×).

**Figure 2:**
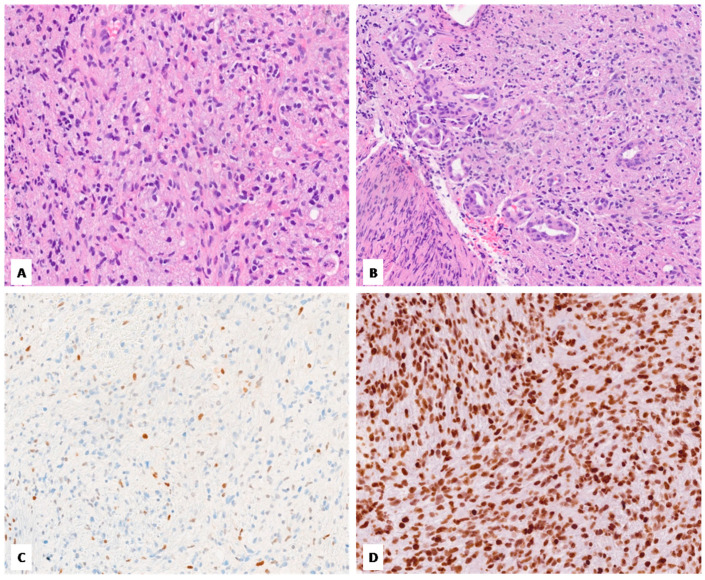
Case no. 2. (**A**) Histological examination showing a moderately cellular, infiltrating glioma with brisk mitotic activity. (**B**) Tumors exhibit rare foci of microvascular proliferation. (**C**,**D**) Immunohistochemically, the lack of immunoreactivity for H3 K27me3 (**C**) and strong and diffuse staining for H3 K27M (**D**) are seen (**A**–**D**, original magnifications 300×).

**Figure 3:**
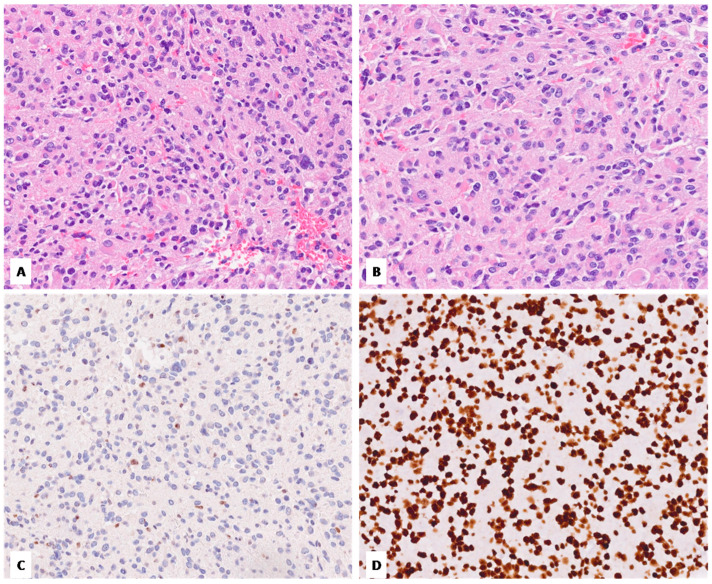
Case no. 3. (**A**) Tumor shows moderate cellularity and mild nuclear pleomorphism. (**B**) Neoplastic cells with a gemistocytic morphology are found within the neoplasm. (**C**,**D**) Additionally, in this case, the immunohistochemical loss of H3 K27me3 (**C**), along with the strong immunoreactivity for H3 K27M (**D**), confirmed the diagnosis of H3 K27-altered DMG (**A**–**D**, original magnifications 300×).

Our cohort included two females and one male. The patient ages at the time of diagnosis ranged from 31 to 77 years, with an average of 46 years. All tumors were located in the thalamus. Figure 1, Figure 2 and Figure 3 show the main histologic and immunohistochemical findings of our series.

All cases exhibited an astrocytic morphology consistent with high-grade glioma; tumor cellularity was moderate in two cases (case no. 2 and no. 3) and high in the remaining case (case no. 1); a gemistocytic component was seen in one case (case no. 3). One tumor (case no. 2) lacked cellular pleomorphism, which was conversely seen in the remaining two cases (mild in case no. 3 and severe with multinucleated giant cell component in case no. 1). Microvascular proliferation was identified in just one case (case no. 2), while necrosis was found in case no. 1; all tumors exhibited brisk mitotic activity. Immunohistochemically, the loss of nuclear staining for H3 K27me3 was observed in all cases, which conversely showed diffuse and strong immunoreactivity for H3 K27M. No cases showed evidence of IDH1 p.R132H mutation by immunohistochemistry. ATRX was retained in all cases, while p53 was overexpressed (>10%) in two cases (case no. 1 and no. 3). The Ki-67 proliferative index was variably high, ranging from 15% to 50%.

Next-generation sequencing analyses, performed as previously described [9], confirmed the diagnosis of H3 K27-altered DMG in all three cases, demonstrating the presence of H3 p.K28M mutation. No additional molecular alterations were found, except for RB1 (p.Arg358Ter) mutation and KIT- PDGFRA copy number gain in case no. 1.

All cases underwent intracranial radiotherapy (50 Gy for 6 weeks). No case showed local recurrence of disease nor leptomeningeal spread at a median follow-up time of 5 months (range 4–13 months).

### 3.2. Literature Review

**Figure 4:**
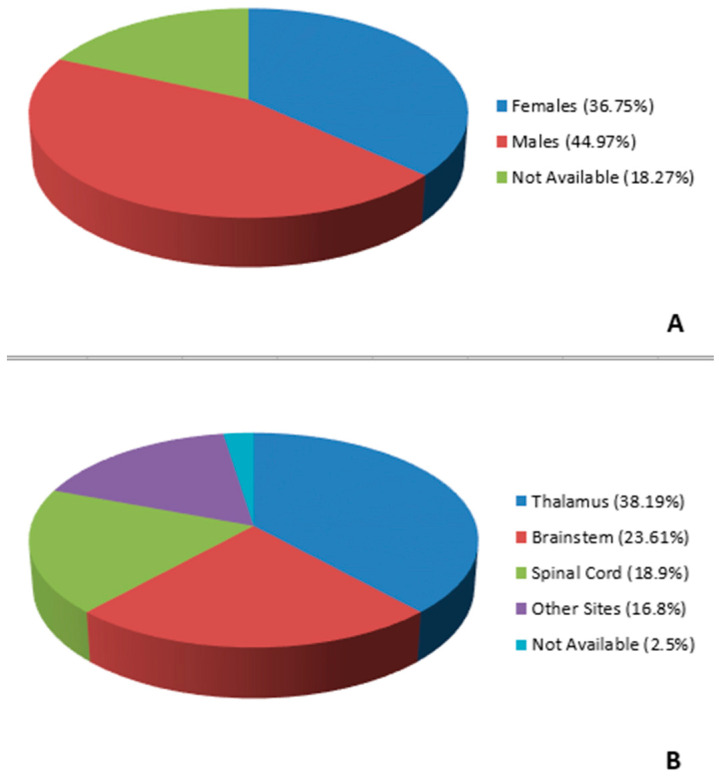
Gender (**A**) and anatomic site (**B**) distributions among adult H3 K27-altered DMGs reported in the literature.

In total, from January 2010 to December 2023, approximately 484 cases of adult patients (over 18 years of age) have been published in the literature [12,13,14,15,16,17,18,19,20,21,22,23,24,25,26,27,28,29,30,31,32,33,34,35,36,37,38,39,40,41] to the best of our knowledge, with an age range between 18 and 83 years. The radiographic, epidemiological, clinical and prognostic data of H3 K27-altered DMGs in adults are not well known. We have summarized all cases of adult tumors (*n* = 487) reported in the literature, including our series, in Table 2. According to the available data, there were 179 females and 219 males (gender was absent in 89 cases) (Figure 4A). The majority of patients were males, and the overall male-to-female ratio was 1.22:1. The date of clinical presentation was not reported in most cases. The age ranged between 18 and 83 years, with a median of 35.5 years. Tumors most frequently occurred in people aged between 18 and 40 years. The three most frequent sites were the thalamus (n = 186; 38.19%), followed by the brainstem (n = 115; 23.61%) and the spinal cord (n = 92; 18.9%) (Figure 4B). Overall, the female subgroup had a median age of 38 years (range 18–83 years), and the male subgroup had a median age of 34 years (range 20–69 years). In both subgroups, the most frequently affected site was the thalamus. The mean follow-up time was 15.6 months and ranged between 3 and 59.2 months. Follow-up data were not available in 133/487 cases (27.31%). Leptomeningeal dissemination was observed in 39 of 164 cases (23.78%), while there was no information on this feature in the remaining 214/487 cases. Data about local recurrence of disease were not available in 171/487 cases, while they were found in the remaining 77/204 (37.75%). For 145 patients, no data regarding the treatment were available. The majority of patients (n = 148; 30.39%) underwent surgery alone, and 79 patients (16.22%) underwent surgery plus adjuvant chemoradiotherapy. Only chemoradiotherapy was administered to 78 patients (16.01%), while chemotherapy alone and radiotherapy alone were administered to 16 (3.28%) and eight patients (1.64%), respectively. Four patients (0.82%) did not undergo any treatments.

## 4. Discussion

Although several studies and case series have tried to describe the radiologic features of adult H3 K27-altered DMGs, they still remain controversial and nonspecific due to their relative rarity and the difficulties in obtaining an accurate integrated diagnosis [6,7]. Adult H3 K27-altered DMGs affect males slightly more frequently than females and patients aged between 18 and 40 years, while they are rarer in people aged > 40 years. They mostly arise from the thalamus followed by the brainstem and the spinal cord, while the brainstem represents the most affected site in children.

Histologically, adult H3 K27-altered DMG are considered WHO grade 4 tumors even if necrosis and/or microvascular proliferation are absent, as their pediatric counterparts. This entity encompasses a wide spectrum of morphological features, mainly reminiscent of high-grade astrocytoma, and no significant differences regarding histologic grade and morphology have been reported between adult and pediatric age groups [6]. It has been reported that H3 K27-altered DMGs lack mitoses, necrosis and microvascular proliferation in approximately 10% of cases [1,5]; in our series, brisk mitotic activity was seen in all cases, while foci of microvascular proliferation and necrosis were found only in case no. 2 and case no. 1, respectively. Some colleagues [14] described a series of 47 cases showing different anatomic locations and a wide morphological spectrum including giant cell features, epithelioid/rhabdoid morphology, primitive neuroectodermal tumor (PNET)-like components, neuropil-like islands, pilomyxoid and ependymal areas, sarcomatous and/or glioneuronal differentiation and areas resembling pleomorphic xanthoastrocytoma. The authors also reported that p53 overexpression and ATRX immunohistochemical loss were frequently shared by tumors harboring H3 p.K28M mutation, with these alterations being more frequently encountered in thalamic and pontine tumors, respectively [14]. In our series, we also found p53 immunohistochemical overexpression (>10%) in two out of three cases, while the nuclear expression of ATRX was retained in all cases. Tu et al. [24] described an unusual case of H3 K27-altered DMG with chondroid metaplasia, exhibiting a high-grade glioma morphology with foci of microvascular proliferation and necrosis and a well-differentiated cartilaginous component.

Although surgical gross total resection represents an ideal treatment for DMGs, it is often not feasible due to the difficulties of the anatomical site, in which it is often difficult to obtain even a valid biopsy for an integrated histological and molecular diagnosis [6,7]. Accordingly, surgery alone (30.39%) represents the most frequent therapeutic approach in adult patients, followed by surgery plus adjuvant chemoradiotherapy (16.22%). In 16.01% of the reported cases, the patients underwent only chemoradiotherapy, while chemotherapy and radiotherapy as sole treatments were reported in a minority of patients. H3 K27-altered DMGs exhibit poor prognosis both in adult and pediatric patients; however, while pediatric median survival ranges from 9 to 12 months, the overall prognosis in adults is less clear, with some studies reporting better outcomes compared to children [6]. It has been reported that adult DMGs tend to be more indolent and have longer survival times than their pediatric counterparts [6,7]. The results of our literature review also demonstrated that adult tumors behave more indolently with long follow-up times (mean value of 15.6 months; range 3–59.2 months) and leptomeningeal dissemination and local recurrence occurring in only 23.78% and 37.75% of cases, respectively. When comparing our cases to the literature on H3 K27-altered DMG in adults, several similarities and differences emerged. Our cases involved patients aged 31, 32 and 77 years. The literature reports cases across a wide age range (18–83 years) with a median of 36.5 years. Two of our cases fell within the more common age group (18–40 years), which is frequently affected according to the literature. All our cases involved the thalamus, which is also the most frequently reported site in the literature (38.19%). This indicates that the thalamus is a preferential site for H3 K27-altered DMG, both in our cases and in published cases. Our cases included a 77-year-old male, which is less commonly seen in the literature in which these tumors predominantly affected younger adults. This finding highlights a demographic difference, namely that H3 K27-altered DMG is typically more prevalent among younger individuals. Case no. 3 occurred in a patient with a history of surgery for a different type of brain tumor (thalamic anaplastic astrocytoma). This differs from many cases in the literature where the initial diagnosis of H3 K27-altered DMG is often the first instance of brain tumor diagnosis, without prior surgical interventions for related conditions. In conclusion, while our cases share important similarities with the published literature regarding tumor location and radiographic features, the inclusion of an elderly patient and a complex clinical history in one case illustrate some notable differences. These variations underscore the heterogeneity and clinical complexity observed in H3 K27-altered DMG, emphasizing the importance of individualized assessment and management strategies in such cases.

By conducting a comparative analysis between adult and pediatric cases of H3 K27-altered DMG, the following data emerged [42,43]: (i) The thalamus is the predominant affected anatomic site in adult cases (38.19%); conversely, pediatric DMGs often arise from the brainstem as the most frequent location. (ii) Histologically, both adult and pediatric H3 K27-altered DMG are categorized as WHO grade 4 tumors. They exhibit high-grade features resembling astrocytomas, although morphological variations such as giant cell features or epithelioid/rhabdoid morphology have been described in both age groups. (iii) Molecular markers such as p53 overexpression and ATRX immunohistochemical loss are reported in DMGs with H3 K27-alterations. Adult cases show variability in these markers (e.g., p53 overexpression in two out of three cases from our series), reflecting the molecular heterogeneity observed across both adult and pediatric populations. (iv) Surgical resection, often challenging due to anatomical constraints, is less frequently achieved in adults compared to children. Adult cases demonstrate variable treatment strategies, predominantly involving surgery with adjuvant therapy or combined chemoradiotherapy, reflecting the complex management decisions in adult H3 K27-altered DMG cases. (v) Prognosis in adult H3 K27-altered DMG remains less defined compared to the generally poor outcomes observed in pediatric patients (median survival 9–12 months). Some studies suggested a relatively indolent course in adults with potentially longer survival times, though outcomes can vary widely.

We believe it may be useful to mention that, differently from diffuse gliomas with H3.3 p.G35R (G34R) or p.G35V (G34V) mutations, which appear to be primarily hemispheric and fulfill the diagnostic criteria for “diffuse hemispheric glioma, H3 G34-mutant”, it has also been reported that some diffuse gliomas with H3 p.K28M mutations combined with the loss of H3 K27me3 may rarely exhibit the lack of involvement of midline structures and do not fulfill the diagnostic criteria for H3 K27-altered DMG [44]. According to the WHO classification recommendations, a diagnosis of “diffuse hemispheric gliomas with H3 p.K28M mutation not elsewhere classified (NEC)” should be rendered for such tumors [44].

## 5. Conclusions

In summary, while both adult and pediatric H3 K27-altered DMGs share common histological and molecular features, significant differences in clinical presentation, radiological characteristics, treatment approaches and prognosis underscore the importance of tailored management strategies. One of the main limitations of this present study lies in the impossibility of performing survival analyses and applying a multivariable Cox model to the data extracted from the literature review, as the data are reported and organized by study cohort rather than at the individual level. In addition, we believe that the sometimes-conflicting data regarding the clinical course of these tumors in adults may be due to often incomplete or non-homogeneous follow-up times reported in the literature; in this regard, the need for international registries for these rare tumors must be emphasized.

Further research is crucial to better understand the distinct clinical behaviors and optimize therapeutic interventions for adult patients with H3 K27-altered DMG.

## Figures and Tables

**Table 1:** Clinico–pathological and immunohistochemical features of the cases from our series.

Cases	Gender	Age (Years)	Anatomic Site	Cellularity	Nuclear Pleomorphism	Mitotic Activity	ATRX	p53	Ki-67	Necrosis	MVP
1	F	31	T	High	Severe	Brisk	Retained	Overexpressed	50%	Present	Absent
2	M	77	T	Moderate	Absent	Brisk	Retained	Not overexpressed	30%	Absent	Present
3	F	32	T	Moderate	Mild	Brisk	Retained	Overexpressed	15%	Absent	Absent

Abbreviations: F, female; M, male, T, thalamus; MVP, microvascular proliferation.

**Table 2:** Cases of adult diffuse midline H3 K27-altered gliomas reported in the literature to date.

Author	Cases (*n*)	Gender	Age (Median; Range)	Anatomic Site	Median Follow-Up	Leptomeningeal Spread	Local Recurrence	Treatment
Aihara et al. [13]	9	Not available	38; 20–46	Thalamus	10.4 months	Not available	Not available	Chemoradiotherapy
Solomon et al. [14]	20	Not available	28.5; 20–65	8 Thalamus 7 Spinal cord 1 Brain stem 4 Other midline sites	Not available	Not available	Not available	Surgery
Feng et al. [15]	43	15 Female 28 Male	32,7; 20–53	15 Thalamus 28 Brain stem	17.4 months	Not available	No	Surgery + Chemoradiotherapy
Kleinschmidt et al. [16]	13	9 Female 4 Male	52; 27–81	7 Thalamus 4 Spinal cord 1 Hypothalamus 1 Brain stem	9.3 months	Yes (1/13 cases)	Yes (2/13 cases)	Not available
Daoud et al. [17]	7	1 Female 6 Male	41; 25–54	5 Brain stem 2 Other midline sites	9 months	Not available	No	4 Chemoradiotherapy 1 Surgery 2 Not available
Wang et al. [18]	35	16 Female 19 Male	Not available	7 Thalamus 10 Spinal cord 11 Brain stem 7 Other midline sites	Not available	Not available	Not available	Not available
He et al. [19]	1	Female	27	Hypothalamus	9 months	Yes	Yes	Surgery + Chemoradiotherapy
Liu et al. [20]	10	4 Female 6 Male	32; 18–54	Thalamus	Not available	Not available	No	Surgery + Chemoradiotherapy
Schreck et al. [21]	18	10 Female 8 Male	38; 30–68	3 Thalamus 2 Spinal cord 6 Brain stem 6 Cerebellum 1 Other midline site	17.6 months	Not available	Not available	13 Chemotherapy 5 Surgery
Ebrahimi et al. [22]	29	10 Female 19 Male	37; 18–73	15 Thalamus 6 Spinal cord 7 Brain stem 1 Cerebellum	4 months	Yes (11/29 cases)	Yes (7/29) cases	Not available
Yekula et al. [23]	1	Male	36	Other midline site	Not available	Yes	Not available	Surgery
Alzoubi et al. [12]	6	1 Female 5 Male	39; 31–52	3 Thalamus 1 Spinal cord 1 Cerebellum 1 Other midline site	3 months	No	Yes (2/6 cases)	2 Radiotherapy 4 Surgery
Chen et al. [24]	1	Female	20	Prepontine cistern	3 months	Yes	Yes	Surgery
Tu et al. [25]	1	Female	56	Medulla oblongata	5 months	No	Yes	Surgery + Chemoradiotherapy
Schulte et al. [26]	60	Not available	32; 18–71	34 Thalamus 10 Spinal cord 5 Brain stem 4 Cerebellum 7 Other midline sites	59.2 months	Not available	Yes (44/60 cases)	16 Surgery 44 Chemoradiotherapy
Dono et al. [27]	9	3 Female 6 Male	38; 23–68	3 Brain stem 1 Pineal gland 2 Thalamus 1 Cerebellum 2 Spinal cord	18 months	Not available	Yes (5/9 cases)	1 Surgery 8 Chemoradiotherapy
Meyronet et al. [28]	21	12 Female 9 Male	32; 18–82	5 Thalamus 6 Spinal cord 5 Brain stem 3 Cerebellum 1 Hypothalamus 1 Pineal region	19.6 months	Yes (1/21 cases)	Not available	1 Surgery 2 Radiotherapy 9 Chemoradiotherapy 3 Chemotherapy 3 Surgery +Chemoradiotherapy 3 None
Qiu et al. [29]	66	26 Female 40 Male	Not available	38 Thalamus 4 Spinal cord 10 Brain stem 1 Hypothalamus 8 Whole brain 3 Corpus callosum 2 Hemispheres	Not available	Yes (8/66 cases)	Not available	Not available
Gu et al. [30]	5	3 Female 2 Male	42; 27–65	Spinal cord	45 months	No	Yes (3/5 cases)	Surgery + Chemoradiotherapy
Yutaka Fujioka et al. [31]	1	Female	66	Thalamus, left hippocampus and frontoparietal lobes	30 months	Yes	No	Chemoradiotherapy
Low JT et al. [32]	1	Female	83	Pons and cerebellum	4.5 months	Yes	No	Chemoradiotherapy
Babarczyey al. [33]	1	Female	73	Spinal cord, medulla, pons, cerebral peduncles	3 months	No	No	Corticosteroid+Empirical antibiotics
Julien Rousseau et al. [34]	1	Female	18	Thalamus	16 months	No	Yes	Radiotherapy + Chemotherapy
Karita et al. [35]	1	Male	26	Spinal cord	4 months	No	No	Surgery
Kraus et al. [36]	1	Male	28	Spinal cord	Not available	No	No	Surgery + Chemoradiotherapy
Yi et al. [37]	25	18 Female 7 Male	39.1; 23–55	Spinal cord	26.4 months	No	Not available	3 Surgery 6 Surgery + Radiotherapy 1 Surgery + Chemotherapy 15 Surgery + Chemoradiotherapy
Zheng et al. [38]	94	42 Female 52 Male	33; 19–71	31 Brainstem 33 Thalamus 9 Spinal cord 21 Other midline sites	10.5 months	Yes (13 cases)	Yes (9 cases)	Surgery
Sugii et al. [39]	2	2 Male	49 and 24	1 Corpus callosum 1 temporal lobe and hypothalamus	9 months and 6 months	No	No	1 Surgery + Chemoradiotherapy 1 Surgery + radiotherapy
Chen et al. [40]	1	Male	32	Spinal cord	6 months	No	No	Radiotherapy
Aftahy et al. [41]	1	Male	24	Spinal cord	3 months	No	Yes	Chemoradiotherapy
Present study	3	2 Female 1 Male	46.6; 31–77	Thalamus	5 months	No	No	Radiotherapy

## Data Availability

All data presented in this article are available from the first author upon reasonable request.
